# Chronic cerebrospinal venous insufficiency in multiple sclerosis: a highly prevalent age-dependent phenomenon

**DOI:** 10.1186/1471-2377-13-20

**Published:** 2013-02-13

**Authors:** Roberta Lanzillo, Marcello Mancini, Raffaele Liuzzi, Orlando Di Donato, Elena Salvatore, Valentina Maglio, Giovanni Vacca, Luca Amato, Gennaro D’Anna, Arturo Brunetti, Vincenzo Brescia Morra

**Affiliations:** 1 Department of Neurological Sciences, Federico II University School of Medicine, Naples, Italy; 2 Institute of Biostructure and Bioimaging, National Research Council of Italy (CNR), Naples, Italy; 3 IRCCS, SDN Foundation, Institute of Diagnostic and Nuclear development, Naples, Italy; 4 Department of Biomorphological and Functional Science, Federico II University School of Medicine, Naples, Italy; 5 Hermitage Capodimonte IDC, Naples, Italy

**Keywords:** Multiple sclerosis, Venous insufficiency, Ultrasonography

## Abstract

**Background:**

This study aimed to investigate the prevalence and clinical relevance of chronic cerebrospinal venous insufficiency (CCSVI) in multiple sclerosis (MS) patients and healthy controls using extra- and intracranial colour Doppler sonography.

**Methods:**

We examined 146 MS patients, presenting with a clinically isolated syndrome, relapsing-remitting, secondary progressive, or primary progressive MS, and 38 healthy controls. Sonographic examination was performed according to Zamboni’s protocol and was performed by three independent sonographers. The results of sonographic examination were compared with clinical and demographic characteristics of the patients.

**Results:**

CCSVI, defined as the presence of at least two positive Zamboni’s criteria, was found in 76% of MS patients and 16% of control subjects. B-mode anomalies of internal jugular veins, such as stenosis, malformed valves, annuli, and septa were the most common lesions detected in MS patients (80.8%) and controls (47.4%). We observed a positive correlation between sonographic diagnosis of CCSVI and the patients’ age (p = 0.003). However, such a correlation was not found in controls (p = 0.635). Notably, no significant correlations were found between sonographic signs of CCSVI and clinical characteristics of MS, except for absent flow in the jugular veins, which was found more often in primary (p<0.005) and secondary (p<0.05) progressive patients compared with non-progressive patients. Absent flow in jugular veins was significantly correlated with patients’ age (p < 0.0001).

**Conclusions:**

Sonographically defined CCSVI is common in MS patients. However, CCSVI appears to be primarily associated with the patient’s age, and poorly correlated with the clinical course of the disease.

## Background

Multiple sclerosis (MS) is an autoimmune disease of the central nervous system (CNS) characterized by inflammation, demyelination, and neurodegeneration [1]. However, neuropathological studies have underlined the presence of a subset of patients with a pattern that closely mimics tissue alterations found in the early stages of white matter ischemia [2]. Moreover, cerebral perfusion studies have shown diffuse hypoperfusion in patients with MS compared with age-matched controls with a greater cerebral blood flow decrease in primary progressive (PP) MS compared with relapsing-remitting (RR) MS [3]. Recently, a strong correlation between MS and a vascular condition called chronic cerebrospinal venous insufficiency was reported [4].

Chronic cerebrospinal venous insufficiency (CCSVI) is characterized by anomalies of the main extracranial cerebrospinal venous routes. CCSVI is detected by selective venography [4-10] and extracranial venous echo-colour Doppler (ECD) [4,9-12] with higher sensitivity and specificity than magnetic resonance venography (MRV) [9]. The prevalence of CCSVI in MS patients is highly variable [4,10,11,13]. The largest study conducted to determine the prevalence of CCSVI in patients with MS, those with clinically isolated syndrome (CIS), those with other neurological diseases, and healthy controls (HCs), using echo-color Doppler (ECD), showed an increased prevalence of CCSVI in MS [14]. This finding suggests that CCSVI does not have a primary causative role in the development of MS. However, perfusion studies have shown a decrease in cerebral perfusion in MS subjects and HCs with CCSVI [15,16].

To date, there are only few studies regarding the relationship between venous abnormalities, phenotype, and the clinical course of MS [17-19], with different and sometimes conflicting results.

This study aimed to investigate the prevalence of CCSVI in a representative MS population and HCs using ECD. We also investigated the clinical relevance and correlations of CCSVI with MS disease parameters.

## Methods

### Patients

This single-centre, cross-sectional study included 171 consecutive MS patients and 41 sex- and age-matched HCs. Inclusion criteria were diagnosis of MS, according to the McDonald criteria [20], including RR, secondary progressive (SP) MS, and PPMS, as defined by Lublin [21], and diagnosis of CIS. The HC group included relatives of MS patients and healthy volunteers. For all patients, exclusion criteria were the presence of relapse and steroid treatment in the 30 days preceding study entry and, for HCs, a history of cerebral congenital vascular malformations and pre-existing medical conditions known to be associated with brain pathology (e.g., cerebrovascular disease and a positive history of alcohol abuse).

### Study assessments

All of the participants underwent a clinical examination and intra- and extracranial ECD of the neck in the same week. Biochemistry was performed to exclude haematological or other medical conditions that could affect haemorheology. Standard demographic and clinical information on all participating subjects were acquired. This included, but was not limited to age, sex, familial history, detailed medical history of vascular risks with particular emphasis on venous diseases, age at disease onset, age at diagnosis, time interval between first and second relapses, annualized relapse rate, and current and previous therapy information. Disease onset symptoms were retrospectively recorded and classified into five categories according to the Expanded Disability Status Scale (EDSS) functional system involved: visual, pyramidal, sensory, subtentorial (cerebellar and brain-stem), and spinal symptoms. Actual disease phenotype was also assessed and divided into the same five categories, according to the most disabled functional system at the time of evaluation. A physical examination was performed with measurement of blood pressure, and EDSS [22] and single EDSS functional systems scores, and Multiple Sclerosis Severity Scale (MSSS) [23] scores were calculated, and disease subtype was classified.

### Standard protocol approvals, registrations, and patient consents

This study was approved by the “Carlo Romano” ethics committee of the Federico II University of Naples, and informed consent was obtained from all subjects.

### Colour Doppler sonography evaluation

Colour Doppler sonography was performed by the same radiologist (MM with greater than 20 years of vascular ultrasound experience) with the iU22 Ultrasound System (Philips, Amsterdam, The Netherlands) equipped with a 3.0–9.0 MHz linear wide-band transducer, a 5.0–8.0 MHz microconvex probe, and a 1.0–5.0 MHz phased array transcranial probe.

Intracranial and extracranial venous outflow were evaluated according to the Zamboni criteria, based on the detection of five criteria as previously described [4]. A subject was considered CCSVI-positive if two or more Zamboni criteria were fulfilled. The radiologist that performed ECD was not blinded to the disease status of patients.

The images were stored and anonymously coded with an identification number. A further evaluation was performed off-line and independently repeated twice by two blinded radiologists (OD, VM) who reviewed all the images. The CCSVI diagnosis was considered valid only if the findings of the three radiologists agreed for each single criterion.

### Statistical analysis

Continuous data are reported as mean and standard deviation or median and range, and categorical data as percentages. For descriptive statistics and estimates of prevalence, the *t*-test, the *U* test (Mann–Whitney), Fisher’s exact test, and the *χ*^2^ test were used. Despite the fact that diagnosis of MS is not a gold standard test to diagnose CCSVI, sensitivity, specificity, and relative odds ratios (ORs) between HCs and patients with MS were calculated, using direct computation from 2 × 2 tables, to obtain results easily comparable with previous studies [4,14]. Prevalence rates for each of the five Zamboni criteria, as well as for different CCSVI presence groups, were calculated. Residual analysis was performed to assess differences in the distribution of these venous criteria among MS subtypes. Logistic regression techniques were used to evaluate correlations between CCSVI presence and clinical parameters. Significance was denoted when p was <0.05 by using two-tailed tests. Statistical analyses were performed using SPSS (version 18.0 Chicago, IL).

## Results

Demographic and clinical data of MS patients and control groups are shown in Table 1. Significant differences in age, EDSS, and disease duration were evident in the subgroups. MS patients belonged to four clinical subtypes (53 RR, 58 SP, 19 PP, and 16 CIS), and were treated in 70% of cases with various therapies (59 with interferon beta-1, 30 with natalizumab, 8 with glatiramer acetate, 3 with fingolimod, 1 with azathioprine, and 1 with mitoxantrone). Of the remaining 44 untreated patients, eight were naïve to any disease modifying therapy, while the others had been previously treated with one or more different drugs.

**Table 1:** Demographic and clinical characteristics of HCs, all MS patients, and MS subtypes

	**Disease group (n)**							
	**HCs (38)**	**All MS (146)**	**P value**	**CIS (16)**	**PPMS (19)**	**RRMS (53)**	**SPMS (58)**	**P value**
Age (y)	37±10	40±9	0.112	32±7	48±11	37±9	43±7	0.001^a^
mean (±SD)								
M/F	18/20	49/97	0.115	6/10	6/13	13/40	17/41	0.001^b^
EDSS		4		2.3	5.5	3.0	5.0	0.001^a^
median (range)		(1.5-7.0)		(1.5-3.5)	(3.5-7.0)	(2.0-5.0)	(2.5-7.0)	
MSSS		5.3		6.3	5.3	5.4	4.9	0.122
median (range)		(1.2-9.5)		(2.1-8.6)	(3.7-9.1)	(1.5-8.6)	(1.6-9.4)	
Disease duration (y)		11		1	13	7	16	0.001^a^
median (range)		(0–35)		(0–11)	(4–24)	(1–29)	(3–35)	
Onset age (y)		28.0		29.3	35.0	27.6	27.0	0.036^c^
median (range)		(13.0-57.0)		(18.5-41.0)	(18–57)	(15–46)	(13–44)	

^a^Age, EDSS, and disease duration were significantly higher in PPMS and SPMS patients than in CIS and RRMS patients.

^b^Male sex was significantly prevalent in PP patients.

^c^Onset age was significantly higher in PPMS patients than in RR and SP patients.

Abbreviations: *CIS*: clinically isolated syndrome; *HCs*: healthy controls; *MS*: multiple sclerosis; *PPMS*: primary progressive multiple sclerosis; *RRMS*: relapsing-remittent multiple sclerosis; *SPMS*: secondary progressive multiple sclerosis.

It was not possible to assess all 5 Zamboni criteria for the absence of an adequate temporal window or for image artefacts that had not allowed full exploration of the jugular valve plane in nine patients (5%). Concordance among the three ultrasonographers on the presence or absence of CCSVI was found in 146/171 (85.4%) MS patients and 38/41 (92.7%) HCs who were included in the statistical analysis.

**Figure 1:**
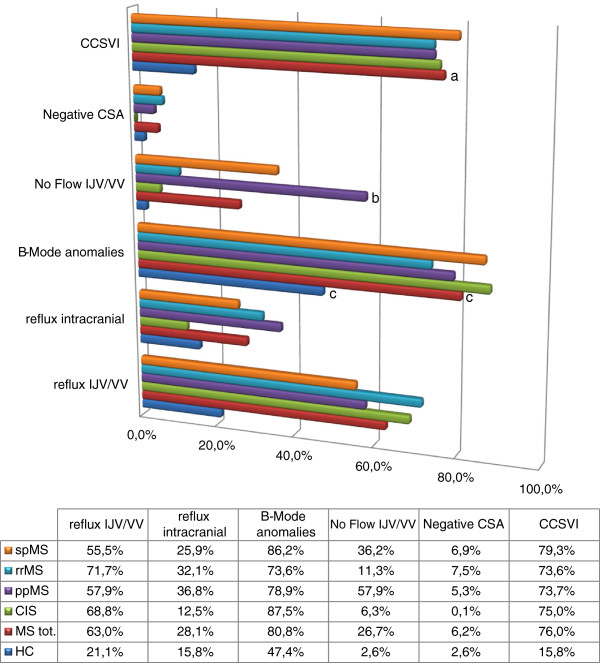
**Prevalence rates of Zamboni criteria and CCSVI for disease group (HC, MS) and MS subtype. **^a^The prevalence of CCSVI was significantly higher in the MS group than in the HC group (p<0.001). ^b^The absence of IJV and/or VV flow (Cr 4) was significantly more frequent in the PPMS form of disease compared with the RRMS form (p<0.005). ^c^B-mode anomalies were the most frequently detected anomalies in MS patients and HCs. Abbreviations: *CIS*: clinically isolated syndrome; *CCSVI*: chronic cerebrospinal venous insufficiency; *HCs*: healthy controls; *MS*: multiple sclerosis; *PPMS*: primary progressive multiple sclerosis; *RRMS*: relapsing-remittent multiple sclerosis; *SPMS*: secondary progressive multiple sclerosis; *Cr*: Echo-Doppler criterion.

One hundred and eleven MS patients (76%) and six HCs (16%) were considered as CCSVI-positive (Figure 1, p<0.001). The sensitivity and specificity of the presence of CCSVI for MS were 76% (95% CI 68.6–82.4) and 84% (95% CI 78.3–93.1), respectively, with an OR of 16.9 (95% CI 6.5–43.8) (Table 2). The prevalence of one or more positive Zamboni criteria was higher in the MS group than in the HC group (93.2% vs 63.2%, p<0.001). For three fulfilled criteria, the prevalence was significantly higher in the MS group (32.2%) than in the HC group (7.9%, p<0.003). The prevalence of five single venous criteria is shown in Figure 1. B-mode abnormalities of the internal jugular vein (IJV) presenting as stenosis, malformed valves, annulus, and septa (criterion 3), were the most frequently detected anomalies in MS patients (80.8%) and HCs (47.4%), followed by reflux in the jugular or vertebral vein (criterion 1) (63.0% in MS and 21.1% in HCs).

**Table 2:** Sensitivity, specificity, and odds ratios of single colour Doppler sonography criteria in MS patients and HCs

	**Sensitivity, % (95% CI)**	**Specificity, % (95% CI)**	**Odds ratio, (95% CI)**
Reflux of the IJV and/or VV (Cr 1)	63,0	78,9	6,4
	(55,0-70,5)	(64,2-89,5)	(2,7-14,9)
Intracranial reflux (Cr 2)	28,1	84,2	2,1
	(21,3-35,8)	(70,3-93,1)	(0,8-5,4)
IJV stenosis B-mode anomalies (Cr 3)	80,8	52,6	4,7
	(73,9-86,6)	(37,1-67,8)	(2,2-10,0)
No flow in the IJV and/or VV (Cr 4)	26,7	97,4	13,5
	(20,0-34,3)	(88,4-99,6)	(1,8-101,6)
Negative CSA (Cr 5)	6,2	97,4	2,4
	(3,1-10,9)	(88,4-99,6)	(0,3-19,8)
CCSVI	76,0	84,2	16,9
	(68,6-82,4)	(70,3-93,1)	(6,5-43,8)

An odds ratio greater than 1.0 indicates an increased likelihood of a diagnosis of CCSVI in MS patients versus controls.

Abbreviations: *MS*: multiple sclerosis; *HCs*: healthy controls; *CCSVI*: chronic cerebrospinal venous insufficiency; *Cr*: criterion; *IJV*: internal jugular vein; *VV*: vertebral vein; *CSA*: cross sectional area; *CI*: confidence interval.

CCSVI was equally distributed in the different MS subtypes (*χ*^2^= 0.583, p=0.90), while the absence of detectable flow in the IJV or in the vertebral vein (VV) (criterion 4) was significantly less frequent in the non-progressive forms compared with the progressive forms of the disease (11.3% in RR and 6.3% in CIS patients, vs 57.9% in PP, p<0.005; and vs 36.2% in SP, p<0.05) (Figure 1). The presence of CCSVI was positively related to age in the whole sample (p=0.001) and in the MS group (p=0.003), but not in HCs (p=0.635). No correlations were found between the presence of CCSVI and sex or clinical parameters, such as age at onset, EDSS, MSSS score, disease duration, number of total relapses, relapses in the last 2 years, and mostly impaired EDSS functional system. Onset symptoms were found in 132 patients (90.4%). No difference in the prevalence of CCSVI was found in relation to disease onset symptoms. Therapy parameters (number of subsequent disease-modifying therapies to which patients had been exposed and the total time of therapy exposure) were not related to the presence of CCSVI.

Single venous criterion 1, 3, and 4 positivity was related to age in SM patients (p=0.029, 0.012, and 0.0001, respectively). In particular, criterion 1 was strongly related to age in RR patients (p=0.006) and criteria 4 in PP patients (p=0.01)

## Discussion

We assessed the presence of CCSVI in MS patients and HCs, with a high interrater concordance (85.4% and 92.7%, respectively). Our study showed a higher prevalence of CCSVI in MS patients and a relatively higher specificity of CCSVI for MS, with a high OR for the diagnosis of MS *vs* HCs. The presence of CCSVI was strongly related to age in the whole sample and in MS patients, without any other clinical correlation. Single venous criteria were also related to age, and the absence of detectable flow in the IJV and/or VV (criterion 4) was more prevalent in PPMS patients compared with the other forms of MS, and was also related to age.

Our results are consistent with other studies that reported an increased prevalence of CCSVI in MS, despite our finding that sensitivity and specificity were lower than previously reported values [4,9,10]. Our finding of a higher prevalence of criterion 4 in PPMS (58%) is also consistent with other studies that showed significantly more MR venography abnormalities in PP patients [24], as well as a longer cerebral circulation time in patients with progressive MS compared with nonprogressive MS [25,26], suggesting a role of hypoperfusion in neurodegeneration.

We did not find any relation of CCSVI with any clinical or demographic parameters, apart from age. These results do not indicate that CCSVI has a primary causative role in MS, as recently suggested in a recent review [27]. Moreover, a strong correlation between CCSVI and age, especially in MS patients, does not favour the hypothesis of the congenital nature of neck vascular alterations. The absence of such a correlation in HCs in our study might be because of the small number of subjects in this group (power=30%). Haemodynamic alterations in neck veins, such as jugular reflux in elderly people, have been described, and interestingly, they are associated with infratentorial white matter hyperintensity [28].

With regard to our finding of the correlation of criterion 4 with progressive forms of disease, the absence of flow in the IJV and/or VV could be related to factors that determine extrinsic compression of neck veins. The IJV passes below the sternocleidomastoid muscle to join the subclavian vein. Therefore, contraction of neck muscles or rotation of the head may result in a compression of these veins with a significant reduction in blood flow, even approaching complete occlusion [29]. The relation between vein biomechanics, surrounding muscle tone and trophism, and the fascial envelope has been demonstrated in the leg [30,31], but not in the IJVs. Moreover, in our study, the examiner paid attention to the inclination of the patient’s head and used an appropriate neck support to avoid hyperextension or rotation of the head. However, it is not possible to exclude with certainty the influence of neck muscles on venous haemodynamics, especially in patients with higher neurological disability. In the current study, progressive patients were older than nonprogressive patients, and criterion 4, similar to criteria 1 and 3, was related to age, confirming that this appears to strongly influence venous hemodynamic alterations in MS. Indeed, PP and SP patients who showed a similar mean age had a comparable prevalence of criterion 4 (p=0.053).

However, it remains unknown if CCSVI is a causal factor or a consequence of MS. Our findings suggest that CCSVI may be a secondary effect rather than a cause of MS, and they indicate that in MS patients, venous valves become less continent with ageing, in association with morphological and functional changes. Many reports have shown the presence of ageing-related increased fibrosis and thickening of the valve leaflets and vein wall [32-36], and decreased compliance of the vein wall [37-39]. Changes in compliance in the vein wall affect venous blood flow, and thickened, stiff leaflets disrupt normal blood flow during the valvular cycle. Several studies have reported hypoperfusion of brain parenchyma in patients with MS, advancing with disease progression [40-43], and it is possible that neck venous anomalies develop secondarily to reduced perfusion. The combination of chronic hypoxia or intermittent hypoxia and haemodynamic factors creates conditions that convert the endothelium of veins to a proinflammatory and procoagulant phenotype [37], which is characterized by the production of plasminogen activator inhibitor-1, release of von Willebrand factor, exposure of P-selectin, secretion of the vasoconstrictor endothelin-1, and production and release of reactive oxygen species. Moreover, hemodynamic forces are important modulators of vascular structure. In rabbits, progressive intimal thickening occurs when shear stress is reduced to subnormal levels with preserved endothelium [44]. There is no evidence of inflammatory infiltrates in neck vein walls in MS [26]. In MS patients, vein walls appear to undergo remodelling of collagen fibres, altering the collagen I/III proportion [26]. The presence of collagen alterations might be due to age-related changes in the vein wall and valves, which have been described in renal veins [33]. In our study, changes corresponding to those found in normal ageing of veins were found in MS patients, and interestingly, this phenomenon was more evident in progressive MS forms where changes in the vein wall and valves caused the most serious hemodynamic consequences. An interesting hypothesis was recently raised in an article suggesting that a neurological process could play a role in CCSVI progression through vasoactive substances (e.g., endothelin-1) or proinflammatory agents, which could act on previously susceptible segments of blood vessels (i.e., malformed vein valves) [45]. This hypothesis would explain the higher prevalence of CCSVI in MS patients and its relation to patients’ age. The same article also suggested a parallel between venous and aortic valves stenosis, which are different entities, but probably share common features. MS patient live in a state of venous stasis that can be attributed to excessive ageing of veins and venules secondary to three elements: 1) chronic inflammation that induces changes in structure and function of the vein wall (in particular, the endothelium); 2) MS-associated alteration in blood flow; and 3) a shift to a hypercoagulable state [46,47]. These changes are located in the trunk wall and the lesions are non-homogeneous and dispersed. The development of new and modern biomedical imaging techniques would be useful for investigating venous valvular pathophysiology in animal models and human subjects.

## Conclusions

In conclusion, our results suggest a higher prevalence of CCSVI in MS patients, which increases with age, possibly more precociously in MS patients than in healthy people, but without any correlation with disability and disease progression. Normal “ageing” of neck veins should be investigated and it should be verified whether reduced cerebral blood flow and cerebral venular chronic inflammatory exposure, both evident in MS, induce venous brain outflow alterations. We believe that to determine the relationship between CCSVI and MS, a more sophisticated assessment is necessary, especially at the beginning of disease and with a longitudinal prospective.

In particular, more refined and quantitative measures, such as blood flow velocity, volume flow, and cerebral circulation times, could be more reliable in assessing the physiological relevance of venous outflow abnormalities.

## Abbreviations

MS: Multiple sclerosis; CCSVI: Chronic cerebrospinal venous insufficiency; ECD: Echo-colour Doppler; HC: Healthy control; RR: Relapsing remittent; SP: Secondary progressive; PP: Primary progressive; CIS: Clinically isolated syndrome; MRV: Magnetic resonance venography; EDSS: Expanded disability status scale; IJV: Internal jugular vein; VV: Vertebral vein.

## Competing interests

The authors declare that they have no competing interests.

## Authors’ contributions

RoL was involved in study concept/study design, data acquisition, data analysis/interpretation, manuscript drafting, literature research, and clinical studies. MM was involved in the study concept/study design, data acquisition, data analysis/interpretation, manuscript drafting, and literature research. RaL performed statistical analysis, drafted the manuscript, and performed literature research. ODD performed data acquisition and literature research. ES performed clinical studies and data acquisition. VM performed data acquisition and literature research. GV and LA performed literature research. GD performed data acquisition and edited the manuscript. AB participated in the study concept/study design. VBM was involved in study concept/study design, data acquisition, data analysis/interpretation, manuscript drafting, literature research, and clinical studies. All authors read and approved the final manuscript.

## Pre-publication history

The pre-publication history for this paper can be accessed here:

http://www.biomedcentral.com/1471-2377/13/20/prepub
